# Exploration of Multiconformers to Extract Information About Structural Deformation Undergone by a Protein Target: Illustration on the Bcl-xL Target

**DOI:** 10.3390/molecules30163355

**Published:** 2025-08-12

**Authors:** Marine Baillif, Eliott Tempez, Anne Badel, Leslie Regad

**Affiliations:** Université Paris Cité, CNRS, Inserm, Unité de Biologie Fonctionnelle et Adaptative F-75013 Paris, France; marine.baillif@u-paris.fr (M.B.); eliott.tempez@etu.u-paris.fr (E.T.); anne.badel@u-paris.fr (A.B.)

**Keywords:** backbone structural variability, structural conservation, Bcl-xL, binding site mapping, ligand-induced conformational change

## Abstract

We previously developed SA-conf, a method designed to quantify backbone structural variability in protein targets. This approach is based on the HMM-SA structural alphabet, which enables efficient and rapid comparison of local backbone conformations across multiple structures of a given target. In this study, SA-conf (version for python2.7) was applied to a dataset of 130 crystallographic chains of Bcl-xL, a protein involved in promoting cell survival. SA-conf quantified and mapped backbone structural variability, revealing the protein’s capacity for conformational rearrangement. Our results showed that while most mutations had minimal impact on backbone conformation, some were associated with long-range structural effects. By jointly analyzing residue flexibility and backbone rearrangements across apo and holo structures, SA-conf identified key regions where the backbone undergoes structural adjustments upon ligand binding. Notably, the α2–α3 region was shown to be a hotspot of structural plasticity, exhibiting ligand-specific conformational signatures. Furthermore, SA-conf enabled the construction of a structural map of the binding site, distinguishing a conserved anchoring core from flexible peripheral regions that contribute to ligand specificity. Overall, this study highlights SA-conf’s capacity to detect conformational changes in protein backbones upon ligand binding and to uncover structural determinants of selective ligand recognition.

## 1. Introduction

Ligand binding is often associated with structural rearrangements of the target protein, reflecting the dynamic nature of molecular recognition. A detailed understanding of such ligand-induced adaptations is essential for elucidating the molecular mechanisms of activation or inhibition, identifying allosteric sites, and guiding the rational design of ligands in structure-based drug discovery. A common approach to detecting and quantifying ligand-induced structural changes is the direct comparison of experimentally determined apo and holo structures of the same protein, as deposited in the Protein Data Bank (PDB) [1]. This strategy provides valuable insights into how ligand interactions alter protein conformation at both the backbone and side chain levels [2,3,4,5,6,7,8]. While side chain rearrangements are generally more pronounced, several studies have shown that the protein backbone also undergoes measurable conformational changes upon ligand binding [2,3,4,5,6,7,8]. For instance, Gutteridge and Thornton (2005) analyzed apo–holo pairs from 60 enzymes and found that approximately 75% of them exhibited Cα-RMSD (Root Mean Square Deviation) values below 1 Å, suggesting subtle but significant backbone rearrangements [9]. Similarly, Brylinski and Skolnick (2008) examined 521 apo–holo pairs and confirmed the prevalence of low–magnitude backbone displacements [10]. Clark et al. (2019) investigated structural deformations in a dataset of 305 proteins including 2369 holo and 1679 apo X-ray structures [11]. They showed that, in 15% of proteins, ligand binding induces significant conformational changes at the backbone level, particularly in the binding site. These structural rearrangements can lead to large-scale deformations in the overall protein fold. Amemiya et al. (2011) quantified that 18% of ligand-induced structural deformations involve domain-level movements, based on a dataset of 839 apo/holo pairs representing 325 distinct proteins [12].

**Figure 1:**
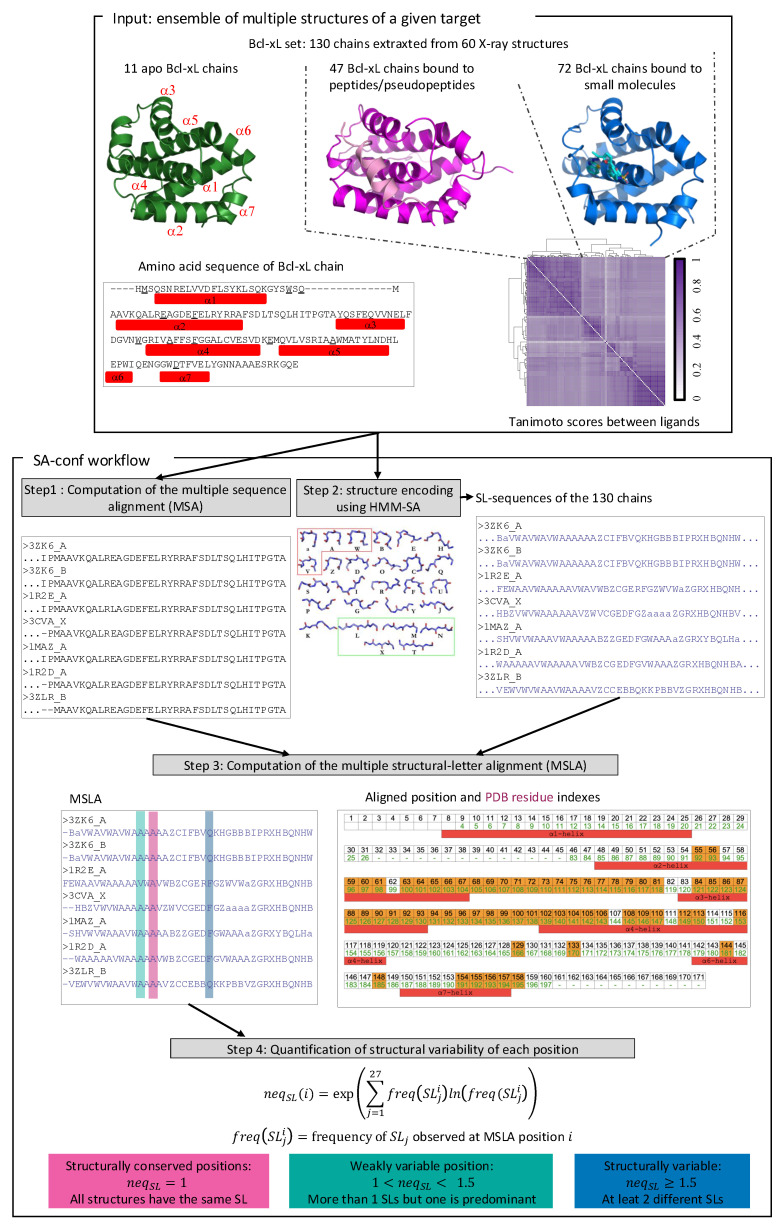
Overview of the SA-conf workflow. SA-conf takes as input a diverse set of structures of the same protein. In this study, it was applied to a dataset of 130 Bcl-xL chains derived from 60 X-ray crystal structures, including 11 apo forms and 119 holo forms complexed with peptides, pseudo-peptides, or small molecules (Appendix A Table A1). To characterize the sequence diversity of the dataset, the aligned amino acid sequence of the wild-type Bcl-xL chain (from the multiple sequence alignment of all chains) is shown. Underlined residues indicate positions with amino acid substitutions across the dataset. Red boxes denote the locations of α-helices. Ligand diversity is illustrated by the matrix of pairwise Tanimoto coefficients computed between co-crystallized ligands, highlighting their chemical similarity. Darker purple squares indicate higher similarity between ligands. The SA-conf pipeline consists of four main steps. Step 1: Extraction of amino acid sequences from all PDB files and computation of a multiple sequence alignment (MSA). Step 2: Encoding of local backbone geometry using the HMM-SA structural alphabet, which transforms each structure into a sequence of structural letters (SLs), each representing the conformation of a four-residue fragment. Step 3: Generation of a multiple structural letter alignment (MSLA) by integrating the MSA with structural encodings. More precisely, SA-conf transforms the MSA into an MSLA by replacing each MSA residue with its corresponding structural letter. A mapping between aligned positions (black) and corresponding residue numbers (green) in the 1MAZ (PDB code) structure is provided. Red boxes mark α-helix positions. Step 4: Quantification of local structural variability using the Shannon entropy exponent ($neq_{SL}$) for each aligned position. Based on the $neq_{SL}$ values, three categories of structural variability are defined: (i) structurally conserved positions ($neq_{SL}=1$), where all structures adopt the same local conformation; (ii) weakly variable positions ($1<neq_{SL}<1.5$), where one conformation dominates with rare deviations; (iii) structurally variable positions ($neq_{SL}\geq 1.5$), where multiple conformations are sampled across the dataset.

To quantify the deformations induced by ligand binding, many studies compare apo and holo structures by calculating the RMSD between them. Despite being widely used, RMSD has several limitations. It provides only a global metric that reflects overall structural deviation, making it inadequate for precisely localizing conformational changes. Moreover, RMSD calculations rely on the superposition of apo and holo structures. To address these limitations, we previously developed SA-conf, a tool that detects backbone conformational variability across sets of protein structures [13] (Figure 1). This method is based on the structural alphabet HMM-SA [14], a library of 27 classes of four-residue Cα fragments defined by their local geometry. HMM-SA simplifies three-dimensional (3D) protein structures by converting them into sequences of structural letters, where each structural letter encodes the geometry of a consecutive four-Cα fragment. From a multiple sequence alignment (MSA), SA-conf generates a structural alignment (MSLA) by replacing each MSA residue with the structural letter corresponding to its local conformation. The exponent of Shannon entropy is used to quantify the variability at each position of the MSA (in terms of amino acids) and MSLA (in terms of structural letters). These variability scores are then used to detect and localize structurally variable regions across multiple protein conformations. By adjusting the composition of the structural dataset (e.g., apo vs. holo, wild-type vs. mutants), SA-conf can pinpoint backbone deformations associated with specific structural or functional determinants. When both apo and holo forms are available, this approach is particularly effective for localizing ligand-induced conformational rearrangements.

In this context, we applied SA-conf to investigate ligand-induced deformations in HIV-2 protease (PR2), a key therapeutic target for HIV-2 infection [15]. The analysis was performed on a dataset of 19 X-ray structures from the PDB, including one apo form and 18 ligand-bound forms. SA-conf revealed that 77% of PR2 residues adopt distinct backbone conformations across the dataset, indicating high structural variability. Further analysis showed that 55% of residues undergo conformational deformations likely attributable to ligand binding. These include residues within the active site, the flap regions, and distal elements such as the α-helix. By characterizing structural variability within the binding site, SA-conf enabled the identification of residues essential for ligand anchoring, as well as those mediating the structural adaptability of PR2 to diverse inhibitors. These findings are essential for elucidating drug resistance, as they show how ligand binding drives structural changes that modulate specificity.

In this study, our objective is to demonstrate the applicability of SA-conf for analyzing structural variability in other therapeutically relevant targets, using Bcl-xL (B-cell lymphoma-extra large) as a case study. Bcl-xL is a key anti-cancer target due to its role in blocking apoptosis. It belongs to the Bcl-2 protein family, which regulates the intrinsic apoptotic pathway. In many cancer cells, Bcl-xL is overexpressed, allowing them to evade cell death by inhibiting pro-apoptotic proteins [16,17]. We apply SA-conf to explore how ligand binding and mutations influence Bcl-xL’s structural variability. To this end, we constructed a dataset of 130 Bcl-xL chains extracted from 60 X-ray structures. These dataset includes both wild-type and mutant forms, as well as apo structures and those bound to various peptides, pseudo-peptides, or small molecules. Applying SA-conf to these dataset revealed both structurally conserved and structurally variable residues. The conserved positions are critical for the protein’s function and stability, while the variable ones adopt distinct conformations across the different structures. By analyzing the rigidity of these variable residues, we identified those that undergo conformational changes upon ligand binding. The exploration of the ligand binding site in terms of variability, flexibility, and ligand-contact conservation enabled us to construct a structural map of the binding site. This analysis revealed three distinct regions within the binding pocket. First, the anchoring subregions, formed by conserved residues that maintain the pocket’s architecture and stabilize ligand binding. Second, central core of the pocket made up of rigid residues, which can nonetheless undergo conformational adjustments upon ligand binding. Third, the peripheral zone composed of flexible and structurally variable residues, which adapt their conformations to accommodate ligand diversity and support binding specificity. Finally, we identified ligand-specific rearrangements in the binding site by analyzing how local residue conformation varies with ligand type. These results highlight the structural adaptability of Bcl-xL to diverse ligands, offering structural insights that may help explain or improve inhibitor selectivity. More broadly, this study demonstrates the value of SA-conf for characterizing binding-induced structural changes and protein–ligand recognition mechanisms.

## 2. Results & Discussion

To investigate the structural variability of Bcl-xL, we assembled a dataset, hereafter referred to as the Bcl-xL set, comprising 130 protein chains extracted from 60 distinct X-ray crystallographic structures of Bcl-xL (Figure 1 and Appendix A Table A1). Among these, 12 chains contain between one and three amino acid substitutions. The dataset encompasses a wide range of ligand types, including 72 small molecules, 35 peptides, and 12 pseudo-peptides, which display substantial variations in molecular size. Specifically, small molecules contain between 19 and 74 heavy atoms, whereas peptides and pseudo-peptides range from 118 to 358 heavy atoms. The ligand set exhibits a moderate level of chemical diversity, as indicated by an average pairwise Tanimoto coefficient of 0.61 ± 0.16 (Figure 1). All co-crystallized ligands bind to the same binding site, located in the groove formed primarily by helices α3, α4, and α5 (Figure 1). Peptides and pseudo-peptides tend to occupy a broader spatial region within the binding pocket compared to small molecules, although significant overlap between ligand types is observed. Given the diversity of the Bcl-xL set—in terms of both protein mutations and ligand types—these dataset provides a robust foundation for exploring the structural variability of Bcl-xL, particularly the conformational changes induced by ligand binding. To this end, we applied the SA-conf tool to analyze and extract local structural variability patterns across the 130 Bcl-xL structures. Figure 1 illustrates the different steps of SA-conf. First, it extracts amino acid sequences from structures and computes a multiple sequence alignment (MSA). Each structure is then encoded as one-dimensional sequence of structural letters using the HMM-SA alphabet, where each structural letter represents the local geometry of a four-residue fragment. The MSA is subsequently converted into a structural alignment by replacing each residue with its associated structural letter. Structural variability is quantified by computing the exponential of Shannon entropy ($neq_{SL}$) at each aligned position. This metric reflects the number of distinct structural letters observed across structures at a given position. A high $neq_{SL}$ value indicates that the position is structurally variable, with a greater diversity of conformations observed across the dataset.

### 2.1. Structural Variability of Bcl-xL: Identification of Conserved and Variable Regions

**Figure 2:**
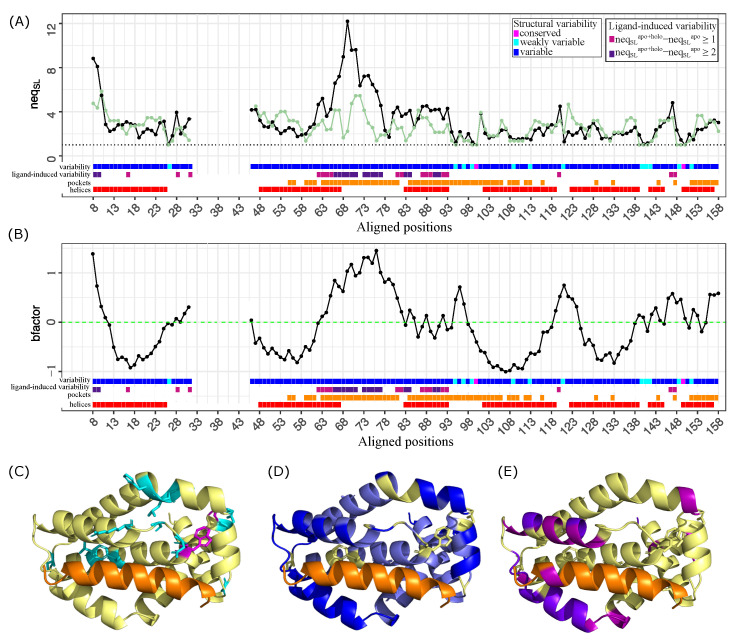
Structural variability and flexibility across the 171 aligned positions of the Bcl-xL dataset. (**A**) Structural variability of Bcl-xL measured by $neq_{SL}$. The black curve shows the $neq_{SL}$ values at each aligned position across the full Bcl-xL dataset, while the green curve corresponds to the apo-only subset. Colored annotation bars below the plot provide additional context. Line 1: Structural variability categories based on the $neq_{SL}$ values from the full dataset. Magenta boxes indicate structurally conserved positions ($neq_{SL}=1$); cyan boxes, weakly variable positions ($1<neq_{SL}<1.5$); blue boxes, variable positions ($neq_{SL}\geq 1.5$). Line 2: Ligand-induced structural variability detected by comparing structural variability ($neq_{SL}$) in the full dataset (apo+holo) to the apo subset. Violet boxes mark positions where $neq_{SL}^{\mathrm{apo}+\mathrm{holo}}-neq_{SL}^{\mathrm{apo}}\geq 1$, indicating moderate ligand-induced variability; purple boxes mark positions with a difference ≥2, indicating substantial conformational change upon ligand binding. Line 3: Positions involved in the consensus binding pocket (orange boxes). Line 4: positions located within α-helices (red boxes). (**B**) Atomic flexibility based on normalized B-factors. Mean normalized B-factor ($B_{norm}$) values are shown at each aligned position, reflecting atomic mobility. The same annotation scheme as in (**A**) is applied below the plot. (**C**,**D**) Mapping of structurally conserved and variable positions onto the 3D structure of Bcl-xL. (**C**) Structurally conserved residues are shown as sticks and colored in magenta; weakly variable residues are shown as sticks and colors in cyan. (**D**) Structurally variable residues are displayed as sticks and colored according to flexibility: rigid (blue), and flexible (dark blue). (**E**) Ligand-induced variability mapped onto the 3D structure. Positions where $neq_{SL}-neq_{SL}^{apo}\geq 1$ are shown in violet and positions where $neq_{SL}-neq_{SL}^{apo}\geq 2$ are colored in purple. (**C**–**E**) The structure shown corresponds to chain A of Bcl-xL complexed with a peptide (PDB code 3R85). Bcl-xL is displayed as cartoon and colored in yellow. Peptide is displayed as cartoon mode and colored in orange.

The Bcl-xL set exhibits an average $neq_{SL}$ of 3.11 (±1.93), indicating a high degree of structural variability. The $neq_{SL}$ value ranges from 1 (e.g., aligned positions 100 and 150) to 12.2 (aligned position 48) (Figure 2A). The greatest variability is observed in the loop connecting helices α2 and α3. Based on the $neq_{SL}$ values, two categories of positions can be defined: (i) structurally conserved positions, where the same conformation is observed in the majority of structures, defined by a $neq_{SL}$ value below 1.5, and (ii) structurally variable positions, which adopt multiple conformations and are characterized by a $neq_{SL}$ value greater than 1.5. In the following sections, we examine these two categories in more detail.

#### 2.1.1. Structural Conserved Positions and Weak Variable Positions

Approximately 8% (14 out of 173) of aligned positions exhibit low structural variability with $neq_{SL}$ values between 1 and 1.5. For example, aligned positions 100 and 150 (corresponding to residues 137 and 187 in the PDB files) display identical local conformations across all 130 chains ($neq_{SL}=1$). Despite the structural heterogeneity of the dataset, these positions consistently adopt the same local conformation across the Bcl-xL structures. This high level of structural conservation across diverse structures suggests strong backbone constraints and a crucial role in maintaining the protein structure and function. This interpretation is further supported by the spatial location of these positions (Figure 2C). For example, aligned position 100 is located within the hydrophobic core formed by W137, I140, W181, I182, W188 and F191—a region known to stabilize Bcl-xL. Other weakly variable positions, including 141, 142, 149, 150, and 152 in the α5-helix, are positioned adjacent to the hydrophobic core, further underscoring their relevance to the stability of the Bcl-xL structure. Several of these weakly variable positions also belong to the ligand binding site, including aligned positions 95, 97, and 99 in the α3–α4 loop, and position 109 in the α4-helix (Figure 2C). Their conservation across structurally diverse complexes suggests that they are essential for preserving the geometry of the binding pocket and facilitating stable ligand interactions. The structural and functional importance of these weakly variable residues is supported by experimental evidence. For example, Feng et al. (2009) showed that the W137A mutation significantly destabilizes the protein structure, confirming the role of W137 in anchoring the C-terminal and BH2 domains to the Bcl-xL core [18]. Manion et al. (2004) demonstrated that mutations E92L (aligned position 55), F97W (position 60), A142L (position 105), and F146L (position 109) alter the binding of Antimycin A, a known Bcl-xL inhibitor [19]. Furthermore, molecular dynamics simulations by Wakui et al. (2018) identified A149 (aligned position 111) and N136 (position 99) as key contact residues involved in interactions with small-molecule ligands [20]. These findings highlight the effectiveness of SA-conf in detecting structurally conserved residues that are crucial, either for protein stability or for ligand recognition.

#### 2.1.2. Structural Variable Positions

In contrast to weakly variable positions, 72% of the aligned positions exhibit strong structural variability, with $neq_{SL}\;\geq \;1.5$. These positions adopt multiple local conformations across the Bcl-xL set. As shown in Figure 2A,D, the N-terminus and the α2–α3 loop (aligned residues 65–80) display particularly high variability with more than four structural letters observed ($neq_{SL}>4$). Given the heterogeneity of the Bcl-xL dataset, the observed structural variability likely reflects a combination of intrinsic flexibility and external factors, including sequence mutations, crystal packing effects, and ligand-induced conformational changes. To better understand how intrinsic flexibility contributes to the structural variability observed across the Bcl-xL dataset, we analyzed variability in relation to residue flexibility, quantified by the average normalized B-factor (Figure 2B). Based on their flexibility, structurally variable positions could be divided into two categories (Figure 2B). Approximately 40% of structurally variable positions are classified as flexible with average normalized B-factor > 0. Their conformational variability appears to be primarily driven by intrinsic flexibility. As shown in Figure 2D, these residues are predominantly located in helices α3, α4, α7, and α8, and the α3–α4 loop. In contrast, 74 structurally variable residues are classified as rigid, with normalized B-factor < 0. They are primarily located in helices α1, α2, α5, and α6 (Figure 2D). Despite their rigidity, these residues adopt distinct conformations across the Bcl-xL structures, indicating that their variability does not result from flexibility. Thus external factors—such as differences in experimental conditions (e.g., crystal form, pH, …), crystal packing, amino acid substitutions or partner binding—could be responsible for the observed structural deformations. These two latter putative sources of structural variability were explored in the following sections.

### 2.2. Amino Acid Substitutions in Bcl-xL Chains Induce Limited Backbone Deformations

We investigated whether amino acid substitutions among Bcl-xL chains contribute to the observed local backbone deformations. As shown in Figure 1, amino acid substitutions were observed at 11 positions across the dataset, yielding 12 mutant structures. Table 1 summarizes these substituted positions, including the distribution of structural letters at each position. One position (aligned position 100) is structurally conserved ($neq_{SL}=1$), while three others (aligned positions 109, 121, and 152) are weakly structurally variable. Six positions (aligned positions 29, 31, 55, 60, 105, and 131) are classified as structurally variable. Table 1 indicates that, for most substituted positions, mutant structures adopt the same local conformation as the majority of wild-type chains. These findings suggest that these mutations, such as E92L, W137A, A142L, F146L, E158K, A168S, and D189A, do not significantly alter the local conformation of the mutated residues. This observation is consistent with previous experimental studies. For example, Feng et al. (2009) showed that the W137A mutation disrupted the architecture of the hydrophobic core, without affecting the overall secondary structure [18]. Structural analyses of the E92L, F97W, A142L, and F146L mutants showed that these amino acid changes did not substantially modify the global structure, with a maximum Cα RMSD of only 0.49 Å observed for the F146L mutant [19].

**Figure A1:**
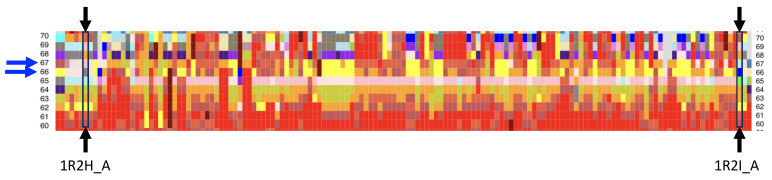
Long-range impact of mutations on Bcl-xL backbone conformation. Structural letter map of the 60–70 (aligned positions in rows) region across the 130 Bcl-xL chains (in columns). Two Bcl-xL mutants are hightlighted. Each case is colored according to the 27 structural letters: [a, A, V, W]-SLs primarily found in α-helices are colored in red, and [L, M, N, T, X]-structural letters primarily found in β-strands are colored in green, other structural letters correspond to loop. For the color scheme of the 27 structural letters, see Figure A2.

In contrast, for the structurally variable positions 29, 31, and 60, the mutant structures do not adopt the most frequent structural letter observed among wild-type chains. However, these differences in conformation do not appear to be mutation-specific (Table 1). As shown in Table 1, even wild-type structures exhibit multiple local conformations at these positions, indicating intrinsic structural variability. For instance, in chain 1R2G_A, an amino acid substitution occurs at variable position 60 ($neq_{SL}$ = 2.59). At this position, chain 1R2G_A adopts structural letter V, which is observed in 19 wild-type chains, while 67% of wild-type chains displays structural letter A. This suggests that the local deformation leading to structural letter V is not a direct consequence of the mutation, but rather reflects natural variability present in the dataset. These observations indicate that the structural changes at these positions are not specifically induced by mutations but are part of the inherent conformational diversity of Bcl-xL. In their study, Manion et al. (2004) reported that the A142L and F146L mutations induced structural changes at residues 101, 104, and 105 (aligned positions 64, 67, and 68), suggesting that some mutations can have longer-range effects on backbone conformation [19]. The analysis of structural letter sequences for aligned positions 60 to 70 in chains 1R2H_A (A142L mutant) and 1R2I_A (F146L mutant) revealed different backbone conformations at aligned positions 66 and 67 (PDB residues 104 and 103) in these two mutant chains, Appendix A Figure A1. These findings illustrate the ability of SA-conf to detect long-range deformations in the protein backbone induced by mutations, even when global structural changes remain limited.

### 2.3. Ligand Binding Induces Localized and Allosteric Structural Variability

In addition to intrinsic or mutation-related variability, ligand binding represents a major source of structural deformation. To assess whether SA-conf captures ligand-induced rearrangements, we compared structural variability in the full Bcl-xL dataset (apo + holo: $neq_{SL}^{apo+holo}$) with that of the apo chains subset (11 chains: $neq_{SL}^{apo}$) (Figure 2A). A total of 71 positions showed increased variability in the complete dataset compared to the apo subset, with 34 of them exhibiting a substantial rise ($neq_{SL}^{apo+holo}-neq_{SL}^{apo}>1$). Notably, aligned positions 68 and 69 (corresponding to PDB residues 105 and 106) were strongly affected by ligand binding. Their $neq_{SL}$ values increasing from 1.65 and 2.23 in the apo subset to 8.98 and 12.20, respectively, in the full set. These results indicate that ligand binding significantly contributes to local backbone deformations at specific positions. Mapping these variable positions onto the Bcl-xL structure revealed that many are located near the peptide-binding site, particularly within the α2–α3 loop (aligned residues 66–77) and the α3-helix (Figure 2E). However, other variable positions—such as aligned positions 120 and 147 (PDB residues 157 and 184)—are located outside the binding site. This suggests that ligand-induced conformational changes are not limited to the binding site but also affect distal regions, suggesting possible allosteric propagation through the protein structure. These findings are consistent with previous studies reporting that small-molecule ligands can trigger substantial rearrangements, particularly within the BH3-binding groove [21,22]. Specifically, structures of Bcl-xL complexed with various BH3 peptides reveal that ligand binding induces an opening of the hydrophobic groove, characterized by a displacement and partial unfolding of helix α3. The extent of these changes varies depending on the peptide partner [23]. Interestingly, 16 aligned positions (11, 19, 21–23, 49, 52–54, 57, 122, 123, 136, 137, 153, and 154) exhibited reduced variability in the full dataset relative to the apo subset. These positions are located within helices α1, α2, α5, and α7 including several that are part of the binding site. These findings suggest that ligand binding can both increase flexibility at certain positions and promote local structural stabilization in specific regions of the protein, including helices distant from the ligand-binding groove. This observation is consistent with previous work by Thébault et al. (2016), which showed that binding of the TCTP BH3 peptide induces elongation of helix α2, reflecting increased local order [24]. Additional studies by Lee et al. (2007), Yang et al. (2012), and Aguirre et al. (2013) also reported reduced backbone flexibility in helix α2 upon peptide binding, further supporting our results [23,25,26]. Overall, SA-conf proves effective in detecting both direct and allosteric backbone rearrangements, capturing the full spectrum of structural responses induced by different ligands. In particular, SA-conf identifies both stabilizing and destabilizing effects of inhibitor binding, reflecting the broad range of structural adaptations that Bcl-xL undergoes in response to diverse molecular partners.

### 2.4. Structural Variability of the Bcl-xL Binding Pocket

**Figure 3:**
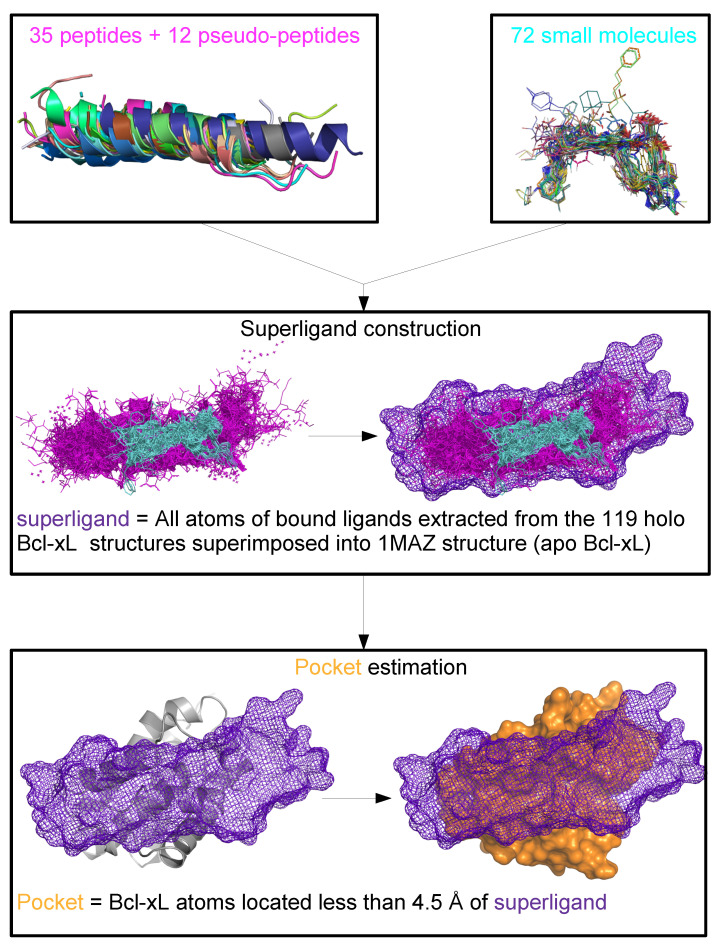
Protocol for estimating the ligand-binding pockets of Bcl-xL using the superligand approach. The superligand method [15], designed to capture ligand chemical diversity and flexibility, was used to estimate the binding pocket of each Bcl-xL chain. A total of 119 co-crystallized ligands comprising 72 small molecules represented as lines), 35 peptides, and 12 pseudo-peptides (represented as cartoon) were extracted from all superimposed Bcl-xL structures. The superligand method involves two steps. Step 1: Superligand construction: The co-crystallized ligands displayed as lines and colored according to ligand type (small molecules in cyan; peptides and pseudo-peptides in magenta) were merged to generate a composite superligand containing 14,110 atoms. The superligand is shown as a mesh and colored according to atom type: peptide and pseudo-peptides atoms were colored in magenta and small molecule atoms were colored in cyan.Step 2: Pocket estimation: The superligand (purple mesh) was positioned within all Bcl-xL chain (gray cartoon) from which the co-crystallized ligand had been removed. The binding pocket of each Bcl-xL chain (apo and holo) is represented as a surface colored in orange and defined as the set of protein atoms located within 4.5 Å of any atom of the superligand (purple mesh).

**Figure 4:**
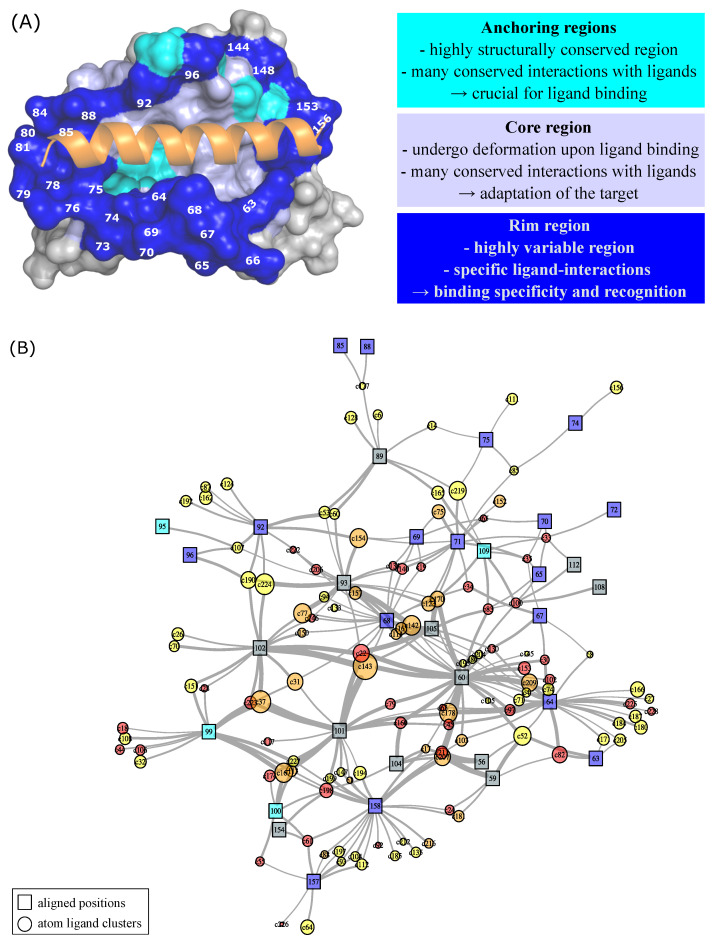
Characterization of the inhibitor-binding pocket of Bcl-xL. (**A**) Structural map of the Bcl-xL inhibitor-binding pocket. The pocket is shown in surface mode and colored in gray and the co-crystallized peptide is displayed as cartoon and colored in orange. The pocket residues are colored based on their structural variability: conserved and weakly variable residues in cyan, rigid variable residues in light blue, and flexible variable residues in blue. Aligned position numbers are indicated. (**B**) Interaction network linking Bcl-xL aligned positions (square nodes) to co-crystallized ligand atom clusters (circle nodes). Square nodes represent aligned Bcl-xL residues and are colored as in panel A. Circle nodes represent ligand atom clusters, defined by spatial proximity across all ligands. Circle size indicates the number of atoms per cluster, reflecting the occupancy frequency of each 3D region by ligand atoms circle node colors indicate the proportion of small-molecule ligands per cluster (ligand-type enrichment): yellow for clusters with <30% small molecules, orange for 30–70%, and red for >70%. Edges represent contacts observed in at least one PDB structure, defined as a residue-ligand atom pair within 4.5 Å. Edge thickness is proportional to the number of structures in which the contact is observed.

To better understand ligand-induced conformational changes, we focused on the structural variability of the Bcl-xL binding site, which plays a central role in molecular recognition and functional regulation. To this end, we extracted ligand-binding pockets from the 130 available Bcl-xL structures using the superligand-based method [15]. In this approach, a pocket is defined as all atoms located within 4.5 Å  of a composite ligand constructed from all co-crystallized molecules (Figure 3 and see Section 3). Using this definition, pockets correspond to the largest region capable of binding any ligand reported to interact with Bcl-xL, regardless of its shape or size, while accounting for both protein and ligand flexibility. This procedure yielded 130 binding pockets, each containing, on average, 54.78 ± 3.33 residues. The consensus Bcl-xL pocket was defined as the union of all residues present in at least one pocket across the entire dataset, resulting in a large pocket with 64 residues. The analysis of the $neq_{SL}$ value of pocket residues showed that the Bcl-xL binding pocket displays substantial structural variability, as quantified by an average $neq_{SL}$ value of 3.61 ± 2.35. Notably, only seven residues were structurally conserved or weakly variable (Figure 2A), whereas the majority (89%) exhibited significant conformational variability (Figure 2A). The structural variability ($neq_{SL}$) and flexibility (normalized B-factors) were projected onto the binding pocket surface, providing a spatial representation of the conformational variability within the binding site (Figure 4A). Interestingly, the central region of the pocket appears relatively rigid and can be subdivided into three distinct subregions. The first two regions includes residues (e.g., aligned residues 99, 100, 109, and 113) that are structurally conserved across all structures, regardless of ligand presence or type. These residues likely form the anchored regions essential for ligand recognition and biological function. The third region, situated at the core of the pocket, contains rigid residues whose conformations vary across structures, likely in response to ligand binding, suggesting induced-fit mechanism. In contrast, the outer rim of the pocket is composed of flexible and structurally variable residues, indicating that these peripheral regions are involved in accommodating ligand diversity and contribute to binding specificity.

To further explore the relationship between ligand binding and conformational variability, we constructed a protein–ligand interaction network (see the Materials and Methods Section). This network connects each Bcl-xL residue to ligand atoms that are located within 4.5 Å in at least one complex structure. Residues were annotated with their $neq_{SL}$ and normalized B-factors to reflect both structural variability and intrinsic flexibility. Analysis of the spatial proximity revealed that central pocket residues—typically rigid but structurally variable—form more numerous and conserved contacts with ligand atoms, particularly with atoms common to multiple ligands (Table 2). In contrast, rim residues, which are both flexible and structurally variable, establish fewer and less conserved contacts. These results suggest that the central region of the pocket is critical for inhibitor anchoring and core binding interactions, while the flexible periphery—mainly composed of helices α2, α3, and the α2–α3 loop—plays a key role in structural adaptation and ligand-specific recognition. Projecting SA-conf results onto the 3D structure of the binding pocket enables the identification of regions essential for ligand anchoring and recognition, as well as those involved in binding specificity.

### 2.5. Ligand-Dependent Conformations in the Binding Pocket

**Figure 5:**
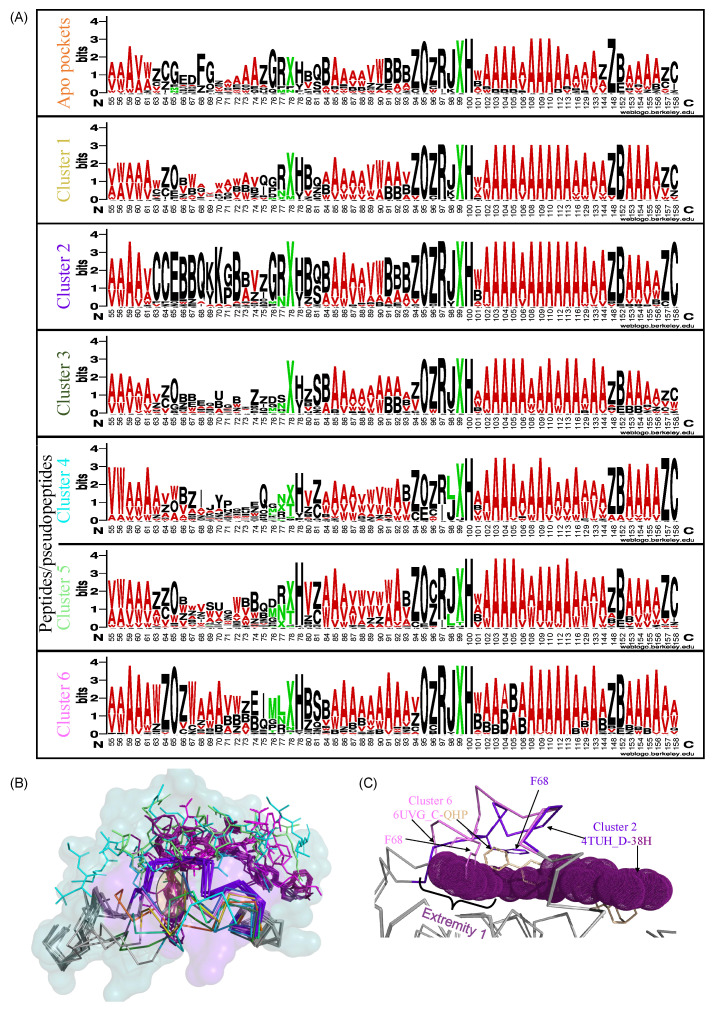
Ligand-induced structural rearrangements in the Bcl-xL binding site. (**A**) Structural letter logos of the Bcl-xL binding site, grouped by ligand clusters. Each column corresponds to an aligned position of the consensus pocket. Stacked letters indicate the relative frequencies of structural letters at that position across all structures. Letter height reflects structural letter frequency, total stack height of the stack reflects structural letter conservation in bits. Structural letters were colored according to the secondary structures: α-helix structural letters in red, β-strand structural letters in green and loop structural letters in black. Logos were generated using WebLogo [27]. (**B**) Structural visualization of the 61–71 (aligned positions) region across the six ligand-binding clusters. The common binding site is displayed in the surface and colored in gray. The 61–71 region is colored according to ligand clusters (see panel (**A**)). Co-crystallized ligands are displayed in stick mode and colored according to ligand clusters. (**C**) Superposition of the binding pockets from two representative complexes: 4TUD (cluster 2) and 6UVG (cluster 6). Proteins are shown in ribbon mode and colored in gray. The 61–71 region is highlighted in violet and pink for 4TUD and 6UVG structures, respectively. The 38H ligand (4TUD) is shown as violet spheres, and the QHP ligand (6UVG) as wheat-colored sticks. Residue Phe68 is displayed in stick mode.

**Figure A2:**
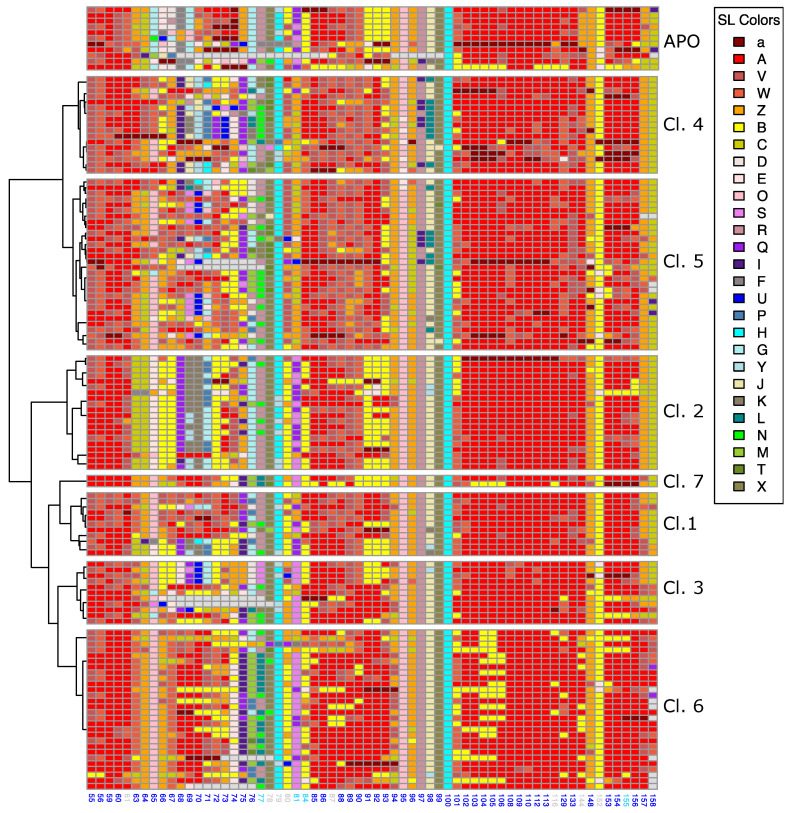
Structural letters of pocket residues. Rows represent Bcl-xL pockets grouped by on the similarity of their co-crystallized ligands. The top 11 rows correspond to apo pockets. Residues are colored based on their frequency across the pocket dataset: residues present in less than 20% of pockets are shown in gray, those present in 20% to 60% in light blue, and those present in more than 60% in dark blue.

To assess whether different ligand chemotypes induce specific structural rearrangements, we analyzed the binding pockets with respect to ligand chemical diversity. Co-crystallized ligands were first classified into six classes based on their physicochemical properties using the Tanimoto coefficient. We then examined the distribution of the 27 structural letters among binding site residues for each ligand class, as shown in Figure 5 and Appendix A Figure A2. This analysis revealed ligand-specific structural signatures within distinct pocket clusters, highlighting how chemically diverse ligands can differentially influence local backbone conformations. For example, peptide-binding pockets (clusters 4 and 5) exhibit specific structural patterns at aligned positions 84 and 85 (PDB residues 121 and 122 on helix α3), with Z or C dominating at position 84 and A and W at position 85. In contrast, apo structures or those complexed with small molecules tend to display S and Q at position 84, and B at position 85. These observations suggest ligand-specific conformational rearrangements at these positions, particularly in response to peptide binding. These findings are consistent with previous observations (Figure 2), which showed increased structural variability at these positions, particularly aligned position 85, in ligand-bound structures. Proximity network analysis (Figure 4B) and structural mapping of the binding pocket (Figure 4A) indicate that these residues are located at the pocket periphery and are rarely in close contact with ligand atoms, suggesting a limited role in direct interactions. Overall, these results support the notion that peptide binding promotes specific structural rearrangements at these peripheral residues, stabilizing local α-helical conformations.

The strongest structural specificities are observed for the region encompassing aligned positions 64 to 77—corresponding to helix α2 and the α2–α3 loop—which exhibits clear ligand-specific structural signatures for some pocket clusters. For example, apo structures display distinct structural letter patterns G..FG....A at aligned positions 65, 68, 69, and 74 (α2–α3 loop). This indicates that ligand binding induces structural rearrangements in this region, consistent with our previous observations that highlighted increased variability at aligned positions 63–72 upon ligand binding. Clusters 2 and 6, on the other hand, show increased structural conservation in the 63–72 region. Specifically, aligned positions 67 and 70 in cluster 6 consistently adopt structural letters W and A, respectively, patterns rarely observed in other ligand clusters. A similar trend is observed in cluster 2, where most pockets exhibit a conserved pattern CCEBBQ[KY]K[GP]B at aligned positions 63–72. Notably, several of these residues—particularly positions 64 and 68—are located in close proximity to numerous ligand atoms (Figure 4B), suggesting a role in ligand accommodation. Thus, the binding of small molecules from clusters 2 and 6 results in specific conformations in the 63–72 aligned positions. At the structural level, this conformation is associated with a shift in the 65–70 aligned positions (Figure 5B). This conformational adaptation appears to be driven by the spatial occupation of the binding site: extremity 1 of cluster 2 ligands extends into a region of the pocket that is not accessed by ligands from other clusters. This is supported by a structural comparison between complexes 4TUH_D–38H (cluster 2) and 6UVG_C–QHP (cluster 6), which reveals a steric clash between aligned residue 68 (PDB residue 105) of 6UVG_C and the extremity of the 38H ligand (Figure 5C). This clash suggests that in cluster 2 complexes, residue 68 undergoes a shift to accommodate the ligand, highlighting the adaptive flexibility of the 63–72 region in supporting ligand-specific binding modes. All these findings are consistent with those obtained using molecular dynamics simulations to demonstrate that the α2–α3 region of Bcl-xL is particularly sensitive to the length and nature of the bound peptide [22,25]. In line with these results, Salam et al. (2018), who compared 24 Bcl-xL–small molecule complexes, also reported substantial conformational changes involving the α2–α3 loop, α3 helix, and flexibility of the α2 helix upon ligand binding [21]. Structural comparisons of Bcl-xL complexes with various peptides, such as Bak BH3, Beclin BH3, and Bim BH3, revealed that the folding of the α2–α3 loop differs significantly depending on the peptide [23,24,28,29,30]. These observations highlight the structural plasticity of different regions of Bcl-xL, particularly the 63–72 region, in response to ligand binding. This demonstrates the role of the region composed of the end of the α2-helix and the α2–α3 loop as a key adaptive element within the Bcl-xL binding site, capable of modulating its conformation to support ligand-specific interactions.

These ligand-specific structural signatures, particularly those identified in the α2–α3 loop region, may have important implications for the rational design of selective inhibitors and reduce off-target effects among Bcl-2 family members. By correlating structural rearrangements with ligand binding affinities, it becomes possible to identify deformation patterns associated with high-affinity binding. Such information could guide the prioritization of compounds that induce favorable conformational states during early-stage screening. In addition, the Bcl-2 family members—Bcl-xL, Bcl-2, Mcl-1, among others—share a highly conserved 3D architecture, particularly at the BH3-binding groove targeted by most inhibitors. A promising approach to improve inhibitor selectivity is to investigate whether certain ligand-induced conformations are preferentially or uniquely adopted by Bcl-xL. Our study reveals such ligand-specific structural signatures in Bcl-xL, particularly in the α2–α3 region. However, it remains unclear whether similar deformations are present in other members of the Bcl-2 family. Future comparative analyses could identify ligands that induce conformational rearrangements that optimize inhibitor binding, but are not observed in other Bcl-2 family members. In this case, the resulting inhibitors would exhibit reduced affinity for these off-targets, improving selectivity. Incorporating these conformational fingerprints into the drug design pipeline offers a rational framework for the development of Bcl-xL-selective inhibitors within this structurally conserved protein family.

Overall, these findings illustrate the ability of SA-conf to capture ligand-specific backbone rearrangements within the Bcl-xL binding pocket, particularly in structurally adaptive regions such as the α2–α3 loop. These observations offer insights into the molecular determinants of ligand recognition and specificity.

## 3. Materials and Methods

### 3.1. Bcl-xL Structure

Due to its central role in inhibiting apoptosis and its overexpression in various cancers, Bcl-xL has emerged as a therapeutically important target for cancer treatment. Structurally, Bcl-xL is composed of eight α-helices (helices α1 to α8) interconnected by flexible loops and includes four conserved BCL-2 homology (BH) domains: BH4 (residues 4–24), BH3 (86–100), BH1 (129–148), and BH2 (180–195), as well as a C-terminal transmembrane domain (210–226) [21], Figure 1. The BH1 and BH2 motifs correspond to loop regions connecting two helices: α4 and α5 for BH1, and α7 and α8 for BH2. The BH3 domain is located entirely on α2 while the BH4 domain is located on α1 and makes a number of stabilizing hydrophobic contacts with α2, α5, and α6. The BH1, BH2, and BH3 domains contribute to the formation of a hydrophobic groove that serves as the binding site for pro-apoptotic BH3-only proteins and inhibitors [31]. This groove is primarily formed by helices α3 and α4, with helix α5 forming its base and additional contributions from residues in the BH2 (α8) and BH3 (α2) domains [32,33].

### 3.2. Dataset Presentation

**Figure A3:**
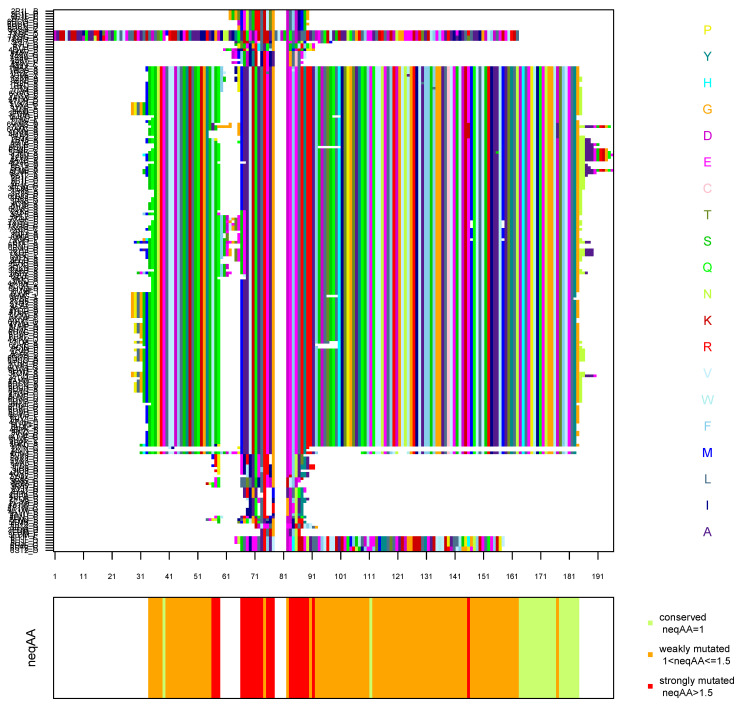
Multiple sequence alignment of the 60 X-ray structures computed by SA-conf.

To investigate the structural variability of Bcl-xL upon ligand binding, we first retrieved 76 X-ray crystallographic structures from PDB associated with the UniProt accession number Q07817. Because SA-conf operates on datasets containing only the protein of interest, a preprocessing step was required to extract Bcl-xL chains from hetero-oligomeric complexes. As an initial step, we ran SA-conf on the complete set of 76 structures. This step identified 797 protein chains, with lengths ranging from 10 to 162 amino acids. Among the 76 structures, 26% were monomeric, 36% dimeric, and 38% multimeric, with two structures (PDB codes 6UVF and 6UVG) containing 12 chains each. From the multiple sequence alignment (MSA) generated by SA-conf (Appendix A Figure A3), we excluded truncated sequences and chains with non-canonical residues, which likely correspond to binding partners rather than Bcl-xL. From homo-oligomeric structures, all Bcl-xL chains were retained to account for potential subtle conformational differences arising from crystal packing, ligand binding, or intrinsic flexibility. Visualization of all selected Bcl-xL chains revealed that six chains exhibit a long α-helix resulting from the fusion of the canonical α5 and α6 helices. This structural rearrangement has been reported in the presence of detergents such as n-octyl-β-D-maltoside or under alkaline conditions (pH 10) [21,34,35,36,37]. As this conformational state does not reflect ligand-induced structural changes, these six chains were excluded from the final dataset. We also excluded hetero-oligomeric complexes where the binding partner occupied the ligand-binding groove to avoid confounding effects in the analysis of ligand-specific binding modes. After this curation process, the final Bcl-xL dataset comprised 130 Bcl-xL chains derived from 60 X-ray structures (Appendix A Table A1), suitable for structural variability analysis using SA-conf.

### 3.3. Co-Crystallized Ligand Classification

To quantify the chemical similarity between co-crystallized ligands, we computed pairwise Tanimoto coefficients using the RDKit cheminformatics library [38]. Ligand structures were extracted from PDB files and converted to RDKit molecule objects using the MolFromPDBFile function. For each ligand, we generated MACCS structural fingerprints (166-bit keys) with the GetMACCSKeysFingerprint function. Tanimoto similarity scores were calculated using the TanimotoSimilarity function from the DataStructs module, based on the MACCS fingerprints. All pairwise scores were rounded to three decimal places and stored in a symmetric similarity matrix. The resulting similarity matrix was used to perform hierarchical clustering with the hclust function in R (version 4.0.2), using the Ward’s minimum variance method (method = “ward.D2”) on a dissimilarity matrix defined as 1—Tanimoto coefficient. Ligands were then grouped into clusters using the cutree function with a fixed number of clusters (k = 7). One of the resulting clusters was a singleton (contained only one ligand) and was excluded from analysis. As a result, six ligand clusters were retained.

### 3.4. Structural Variability Quantification Using the SA-conf Tool

SA-conf is a tool designed to quantify the backbone structural variability of a target protein from a set of its 3D structures. Figure 1 summarizes the main steps of the SA-conf workflow. First, SA-conf extracts the amino acid sequence of each chain from all structure files and computes a MSA using software ClustalW [39].

Second, SA-conf extracts local structures from each chain using the HMM-SA structural alphabet [14,40]. HMM-SA is a classification of four-Cα fragments based on the fragment geometry established by hidden Markov models. In HMM-SA, fragment classes are denoted by structural letters and they are labeled [a, A–Z]. SA-conf simplifies each 3D structure of *p* residues into a sequence of (p−3) structural-letter sequences, where each structural letter corresponds to the fold of a 4-Cα fragment. Each structural letter is assigned to the third residue of the 4-α fragment.

Third, SA-conf transforms the MSA into a multiple structural alignment (MSLA) by replacing each MSA residue with its corresponding structural letter. The distribution of structural letters at each MSLA position provides a direct measure of local structural variability. Aligned positions that exhibit the same structural letter across all chains are considered structurally conserved, indicating a shared local conformation regardless of mutations, binding partners, or experimental conditions. In contrast, positions with different structural letters across chains are classified as structurally variable, highlighting structural variability. To quantify the structural variability within the dataset, SA-conf calculates the exponent of the Shannon entropy ($neq_{SL}$) at each MSLA position (Figure 1). This metric, which ranges from 1 (fully conserved) to 27 (highly variable), represents the effective number of distinct structural letters observed, with higher values indicating greater structural variability. Based on the $neq_{SL}$ values, aligned positions are classified into three categories:

- The structurally conserved positions ($neq_{SL}$ = 1). For these positions, all structures share the same local conformation.
- The weakly variable positions ($1<neq_{SL}<1.5$). For these positions, one structural letter predominates. These positions exhibit rare local conformational changes across structures.
- The structurally variable positions ($neq_{SL}\geq 1.5$). Multiple structural letters are observed at these positions, indicating distinct conformations across chains.

### 3.5. Quantification of Residue Flexibility

To evaluate residue flexibility across Bcl-xL structures, we extracted the B-factor value, also referred to as temperature factors or atomic displacement parameters, for each Cα atom from the corresponding PDB files. These values quantify the degree of isotropic displacement of atomic electron density around its mean position, thereby providing a proxy for atomic mobility [41]. Because raw B-factor values vary across structures due to differences in experimental conditions (e.g., resolution, temperature, and refinement protocols), we normalized the values to ensure consistent comparisons. For each residue *i* in structure *j*, the normalized B-factor, denoted $B_{norm}\left(i,j\right)$, was calculated using the following equation:

$$B_{norm}\left(i,j\right)=\frac{B\left(i,j\right)-\langle B_{j}\rangle }{\sigma_{B_{j}}}$$

where B(i,j) represents the raw B-factor of the Cα atom of residue *i* in structure *j*, and $\langle B_{j}\rangle$ and $\sigma_{B_{j}}$ correspond to the mean and standard deviation of Cα B-factors within structure *j*, respectively. An average $B_{norm}$ value was then computed for each aligned position across the 130 Bcl-xL structures. Positions with an average $B_{norm}$ value greater than zero were classified as flexible, whereas those with values below zero were designated as rigid.

### 3.6. Binding Site Estimation

The ligand binding site of each Bcl-xL chain was estimated using the superligand method [15], presented in Figure 3. This method was chosen for its ability to account for ligand diversity in terms of size, type (small molecules, peptides, and pseudo-peptides), and flexibility. In this approach, the binding pocket is defined as the larger region of Bcl-xL capable to bind any ligand observed in the dataset. The method begins with the construction of the superligand. To do so, each Bcl-xL chain was superimposed into the arbitrarily selected reference chain 1MAZ_A. From the superimposed complexes, a total of 119 ligands were extracted: 72 small molecules, 35 peptides, and 12 pseudo-peptides. The superligand was generated by merging the 119 superimposed ligands. Therefore, this composite ligand reflects the structural diversity and flexibility of all ligands known to bind Bcl-xL. The resulting superligand comprises 14,110 atoms. The next step consisted in estimating the binding pocket from the 3D structures. To this end, the superligand was positioned within the 130 aligned Bcl-xL chains (apo and holo). Each pocket was defined as all Bcl-xL atoms located within 4.5 Å of the superligand (Figure 3). Using this method, 130 pockets were extracted, containing on average 334.01 ± 19.84 atoms and 54.78 ± 3.33 residues.

### 3.7. Protein–Ligand Network

To investigate the relationship between structural variability and ligand binding in Bcl-xL, we constructed a spatial proximity network connecting Bcl-xL residues to co-crystallized ligand atoms, based on their spatial proximity. The construction of this network involved three main steps, as detailed below.

#### 3.7.1. Step 1: Clustering of Ligand Atoms Based on Their 3D Proximity

This step aimed to identify recurrent spatial regions occupied by chemically diverse ligand atoms. We grouped the 14,110 atoms from the 119 co-crystallized ligands based on their 3D coordinates. To do so, k-means clustering was applied to the XYZ coordinates of all ligand atoms. To determine the optimal number of clusters, we evaluated 21 k-means clustering runs with cluster counts ranging from 200 to 400 (in increments of 10). Each k-means run was performed using the kmeans function in R (version 4.0.2), with a maximum of 50 iterations and 50 random initializations to ensure convergence and robustness of the clustering results. Cluster number selection was guided by two criteria:

- Intra-cluster variability: Clusters with the lowest internal variance were favored.
- Ligand atom uniqueness per protein: Each cluster should ideally contain only one ligand atom per Bcl-xL structure, to preserve one-to-one mapping at the complex level.

Based on these criteria, 230 clusters were selected as optimal. Final clustering was then performed using 230 centroids, with 500 repetitions and a maximum of 100 iterations per run. This resulted in 230 clusters of ligand atoms, each grouping together spatially proximal atoms from different ligands. Cluster size ranged from 7 to 136 atoms, with a mean of 48 ± 21.56 atoms per clusters. Highly populated clusters likely represent spatial regions that are frequently occupied by atoms from chemically diverse ligands, suggesting common spatial patterns. In contrast, sparsely populated clusters correspond to regions less consistently occupied across the ligand and more ligand-specific regions.

#### 3.7.2. Step 2: Identification of Potential Protein–Ligand Contacts

For each Bcl-xL–ligand complex, all protein residues within 4.5 Å of any ligand atom, were considered as potential contact residues. These residues were labeled using the corresponding aligned position. Each potential contact was annotated with four attributes: (i) the PDB code of the complex; (ii) the residue identifier (aligned position number_chain); (iii) the ligand atom identifier (atom number_chain); (iv) the atom cluster ID (as defined in Step 1). This procedure identified 11,659 residue–ligand contacts. To enable comparison across the full set of complexes, we replaced ligand atom identifiers with their corresponding cluster IDs. This reduced the dataset to 814 unique contacts, for which we recorded their frequency across the dataset. Only the 284 contacts present in over 10% of complexes were retained for network construction.

#### 3.7.3. Step 3: Network Construction

The final protein–ligand interaction network was constructred using the igraph library [42] in R (version 4.0.2), based on the 284 retained contacts. In this network, nodes represent either Bcl-xL aligned positions or ligand–atom clusters. An edge was established between a residue and a ligand-atom cluster if at least one atom in that cluster was located within 4.5 Å of the residue in at least one structure.

#### 3.7.4. Step 4: Network Analysis

We analyzed the protein–ligand network with respect to the structural variability and flexibility of residues. For each residue, we defined the average number of links that it established. A high average number of links suggests that a residue frequently interacts with ligand atoms located in multiple spatial regions, potentially reflecting a significant role in ligand binding. Conversely, residues with a low average number of links tend to be less involved in ligand binding, suggesting a limited or marginal contribution to protein–ligand interactions. We also computed the average link conservation, considering both the protein and ligand levels. Specifically, we computed (i) the average percentage of structures having the link and (ii) the average size of the ligand–atom cluster that established the link. The first criterion reflects how consistently a residue is involved in ligand contacts across the dataset—thus describing its conservation at the protein level. The average size of the ligand–atom cluster reflects how many distinct ligands contribute to a given link, thus indicating conservation on the ligand level. These metrics reflect how frequently a specific residue–ligand spatial association occurs across the set of Bcl-xL–ligand complexes. A high average percentage of structures with a given link suggests that the residue is frequently engaged in contacts with ligands, pointing to a conserved anchoring role across different complexes. Conversely, a low value indicates a contact dependent on the ligand. Similarly, a link involving a large ligand–atom cluster indicates that many ligands establish contact with the same residue, suggesting a common and potentially functionally important binding hotspot. In contrast, links involving small clusters likely reflect less conserved, more ligand-specific contacts.

## 4. Conclusions

In this study, we demonstrated the capacity of SA-conf to characterize structural variability within large and diverse ensembles of protein structures. Based on a structural alphabet representation of local backbone geometry, SA-conf quantifies position-specific conformational variability independently of global alignment. Compared to traditional methods such as RMSD, SA-conf is more effective at detecting small-scale local conformational changes, even in structurally rigid regions or areas with low-resolution data. Its entropy-derived metric, $neq_{SL}$, provides a fine-grained measure of the number of conformational substates sampled at each position, making it particularly well suited for detecting structural adaptations driven by intrinsic flexibility, point mutations, or ligand binding. SA-conf also handles large and heterogeneous datasets, enabling comparative analyses across multiple conditions. While it does not account for side-chain variability, SA-conf provides a powerful and robust framework for capturing backbone-level dynamics critical for function.

We applied SA-conf to 130 Bcl-xL chains including multiple liganded and unliganded structures, wild-type and mutant forms. Our analysis revealed that Bcl-xL displays a high degree of structural plasticity, with 72% of positions highlighting conformational variability of Bcl-xL. Nevertheless, structurally conserved residues were clearly identified, particularly those involved in maintaining the protein fold—such as PDB residues 137 and 187—or forming conserved interactions within the binding pocket–such as PDB residues 92, 97, 143, and 146—which are critical for ligand anchoring. The conformational variability detected in Bcl-xL can arise from multiple sources, including intrinsic flexibility, amino acid substitutions, and ligand binding. To explore these contributions, we analyzed specific subsets of the dataset using SA-conf to assess how each factor affects local and long-range structural changes. Our analysis showed that the observed substitutions did not induce significant local deformation of the protein backbone at the mutation sites themselves. However, some of these mutations—such as A142L and F146L—were associated with long-range structural rearrangements, indicating that mutations can trigger distal conformational changes despite local rigidity. Most notably, ligand binding was associated with significant conformational rearrangements, both local and distal to the binding site, suggesting allosteric propagation of structural changes. Within the binding pocket, we observed both local rigidification (e.g., in α1, α2, and α5) and more ligand-specific rearrangements, particularly in the α2–α3 loop and α3 helix. Our results revealed a spatial organization of the pocket into three zones: a conserved core with rigid anchor residues, a central region with moderate plasticity contributing to binding stability, and a flexible periphery modulating ligand specificity. This structural insight has direct implications for structure-based drug design, emphasizing which regions may be targeted for high affinity versus selectivity.

Altogether, this work underscores the relevance and effectiveness of SA-conf in exploring conformational diversity across protein ensembles. The Bcl-xL case study illustrates how such analysis can reveal key structural features underlying protein function and ligand recognition, and guide future efforts in rational drug design. Beyond Bcl-xL, SA-conf could be applied to other structurally diverse protein families to identify ligand-induced and mutation-driven conformational signatures. Such insights could support the rational design of selective inhibitors by targeting structural states associated with functionally relevant or disease-related conformations.

## Figures and Tables

**Table 1:** Description of the substituted positions the in Bcl-xL set. For each aligned position affected by at least one amino acid substitution, the following information is provided: column Aligned position: the position index in the MSLA; column PDB residue: the corresponding residue number in the reference structure 1MAZ; column Position category: structural variability classification based on $neq_{SL}$ values (conserved, weakly variable, or variable); column Substitution: amino acid change(s) observed at this position; column SL in the substituted sequence: Structural letter observed at the substitution site for each mutated chain; column SL distribution: frequency of each structural letter observed across Bcl-xL chains at this position. The most frequent structural letter is highlighted in bold, indicating the predominant local conformation.

Aligned Position	PDB Residue	Position Category	Substitution	SL in Substituted Sequences	SL Distribution
6	-	/	M → H	7XGG_F: N	/
29	24	Structurally variable	W → A	6VWC_A: N	E: 1 F: 3 G: 16 I: 1 K: 1
				6VWC_B: NA	M: 1 N: 1 **P: 118**
				7LH7_A: X	X: 1 NA: 6
31	26	Structurally variable	Q → G	7LH7_A: U	A: 1 B: 17 D: 2 E: 10 **H: 92** O: 1 P: 3 U: 1 X: 1 Y: 6 Z: 2 NA: 13
55	92	Structurally variable	E → L	1R2E_A: A	a: 2 **A: 69** B: 1 V: 71 W: 6
60	97	Structurally variable	F → W	1R2G_A: V	a: 2 **A: 100** B: 2 V: 20 W: 25
100	137	Structurally conserved	W → A	3CVA_X: H	**H: 149**
105	142	Structurally variable	A → L	1R2H_A: A	a: 8 **A: 122** B: 18 V: 1
109	146	Weakly structurally variable	F → L	1R2I_A: A	a: 4 **A: 136** V: 8 W: 1
121	158	Weakly structurally variable	E → K	4QVX_A: D	A: 1 **D: 142** H: 1 V: 1 W: 4
				4QVX_B: D	
				6VWC_A: D	
				6VWC_B: D	
				7LH7_A: D	
				7LH7_B: D	
131	168	Structurally variable	A → S	5FMK_A: A	a: 1 **A: 128** B: 3 V: 14 W: 3
152	189	Weakly structurally variable	D → A	4QVX_A: B	a: 2 **B: 133** E: 14
				4QVX_B: B	
				6VWC_B: B	
				6VWC_A: B	
				7LH7_A: B	
				7LH7_B: B	

**Table 2:** Analysis of the protein–ligand interaction network. Column 1: Residue category based on structural variability. Column 2: Average number of connections (edges) formed by a residue within the contact network. Column 3: Average frequency of residue–ligand cluster contact across the Bcl-xL structures. A contact is defined as a residue with at least one atom within 4.5 Å of a co-crystallized ligand atom. Column 4: Average size of ligand atom clusters connected to each residue in the interaction network, reflecting the spatial recurrence of ligand occupation. Standard deviations are shown in parentheses.

Aligned Position Type	Average Number of Links	Average Conservation of Link	Average Size of Ligand Clusters
Rigid and structurally variable positions	9.83 (8.8)	0.26 (0.05)	66.64 (9.58)
Flexible and structurally variable positions	8.18 (8.41)	0.16 (0.03)	54.64 (11.75)
Structurally conserved positions	6.75 (4.92)	0.22 (0.03)	62.79 (4.89)

## Data Availability

The datasets supporting the findings of this study are available as Supplementary Materials and through public repositories. The list of the 130 Bcl-xL chains used in the analysis is provided in Supplementary Table S1 and can be downloaded from the following address: https://owncloud.rpbs.univ-paris-diderot.fr/owncloud/index.php/s/4ftv3OM669K037i, accessed on 6 August 2025. The alignedPosition_description.xls file, describing the aligned positions of the Bcl-xL set is available and can be downloaded from the following address: https://owncloud.rpbs.univ-paris-diderot.fr/owncloud/index.php/s/4ftv3OM669K037i, accessed on 6 August 2025. These files, respectively, contain the PDB identifiers with ligand types and the aligned position annotations (e.g., $neq_{SL}$, POCKET, HELICES, B-factor normalization, and flexibility classification). The SA-conf software executables used in this study is openly available at: https://owncloud.rpbs.univ-paris-diderot.fr/owncloud/index.php/s/l31XQ2TN0beo1py, accessed on 6 August 2025.

## Supplementary Materials

The following supporting information can be downloaded at https://owncloud.rpbs.univ-paris-diderot.fr/owncloud/index.php/s/4ftv3OM669K037i, accessed on 6 August 2025.

## Abbreviations

The following abbreviations are used in this manuscript:

Bcl-xL	B-cell lymphoma-extra large
BH	Bcl-2 homology
Bnorm	Normalized B-factor
Cα	Carbon α
HMM-SA	Hidden Markov Model-Structural Alphabet
MSLA	Multiple Sequence Letter Alignment
MSA	Multiple Sequence Alignment
PDB	Protein Data Bank
PR2	HIV-2 Protease
RMSD	Root Mean Square Deviation
SL	Structural letters
X-ray	Crystallographic
3D	Three-dimensional

## Appendix A

### Appendix A.1. Composition of the Bcl-xL Data Set

**Table A1:** List of the 130 chains comprising in the Bcl-xL dataset. The PDB column indicates the PDB code and the corresponding chain representing Bcl-xL. The ligand column specifies the type of co-crystallized ligand with each Bcl-xL chain: Apo denotes the absence of a ligand, SM refers to a small molecule, P to a peptide, and pP to a pseudo-peptide.

PDB	Ligand	PDB	Ligand	PDB	Ligand	PDB	Ligand
1MAZ_A	Apo	3R85_D	P	4TUH_F	SM	6UVF_G	SM
1R2D_A	Apo	3SP7_A	SM	4TUH_G	SM	6UVF_H	SM
1R2E_A	Apo	3SPF_A	SM	4TUH_H	SM	6UVF_I	SM
1R2G_A	Apo	3ZK6_A	SM	4Z9V_A	P	6UVF_J	SM
1R2H_A	Apo	3ZLN_A	SM	4Z9V_B	P	6UVF_K	SM
1R2I_A	Apo	3ZLO_A	SM	5B1Z_A	P	6UVF_L	SM
2B48_A	Apo	3ZLR_A	SM	5B1Z_B	P	6UVG_A	SM
2P1L_A	P	3ZLR_B	SM	5C3G_A	P	6UVG_B	SM
2P1L_C	P	4A1U_A	pP	5FMJ_A	P	6UVG_C	SM
2P1L_E	P	4A1W_A	pP	5FMK_A	P	6UVG_D	SM
2P1L_G	P	4A1W_B	pP	5VX3_A	P	6UVG_E	SM
2YJ1_A	pP	4A1W_C	pP	5VX3_C	P	6UVG_F	SM
2YJ1_C	pP	4A1W_D	pP	5VX3_E	P	6UVG_G	SM
2YQ6_A	P	4BPK_A	pP	5VX3_G	P	6UVG_H	SM
2YQ7_A	P	4BPK_B	pP	6DCN_A	P	6UVG_I	SM
2YXJ_A	SM	4C52_A	SM	6DCN_B	P	6UVG_J	SM
2YXJ_B	SM	4C52_B	SM	6DCO_A	P	6UVG_K	SM
3CVA_X	Apo	4C5D_A	SM	6DCO_B	P	6UVG_L	SM
3FDL_A	P	4C5D_B	SM	6RNU_A	SM	6UVH_A	SM
3FDM_A	pP	4CIN_A	P	6RNU_B	SM	6UVH_B	SM
3FDM_B	pP	4CIN_B	P	6UVC_A	SM	6UVH_C	SM
3FDM_C	pP	4EHR_A	SM	6UVC_B	SM	6UVH_D	SM
3INQ_A	SM	4HNJ_B	Apo	6UVD_A	SM	6VWC_A	SM
3INQ_B	SM	4PPI_A	Apo	6UVD_B	SM	6VWC_B	SM
3IO8_A	P	4QVE_A	P	6UVE_A	SM	6YLI_A	P
3IO8_C	P	4QVF_A	P	6UVE_B	SM	7CA4_A	Apo
3PL7_A	P	4QVX_A	SM	6UVE_C	SM	7JGV_A	SM
3PL7_B	P	4QVX_B	SM	6UVF_A	SM	7JGV_B	SM
3QKD_A	SM	4TUH_A	SM	6UVF_B	SM	7JGW_A	SM
3QKD_B	SM	4TUH_B	SM	6UVF_C	SM	7LH7_A	SM
3R85_A	P	4TUH_C	SM	6UVF_D	SM	7LH7_B	SM
3R85_B	P	4TUH_D	SM	6UVF_E	SM		
3R85_C	P	4TUH_E	SM	6UVF_F	SM		
